# IDENTIFYING OPPORTUNITIES TO OPTIMIZE PERSON-CENTERED CARE IN LONG-TERM CARE

**DOI:** 10.1093/geroni/igae098.3167

**Published:** 2024-12-31

**Authors:** Tonya Roberts, Jingxi Li, Justin Boutilier, Yonatan Mintz

**Affiliations:** University of Wisconsin - Madison, Madison, Wisconsin, United States; University of Wisconsin Madison, Madison, Wisconsin, United States; University of Wisconsin Madison, Madison, Wisconsin, United States; University of Wisconsin Madison, Madison, Wisconsin, United States

## Abstract

Person-centered care (PCC), or care consistent with preferences, improves long-term care (LTC) resident quality of life, family satisfaction, and staff job satisfaction. Despite benefits, PCC has not been fully adopted across homes. One barrier is a lack of knowledge regarding patterns in resident preferences that can meaningfully inform operational decision-making to improve PCC. This study aimed to take the first step toward filling that gap by: 1) describing LTC residents’ preferences; 2) describing the impact of aligning care with preferences, and 3) identifying gaps in care that could be improved with operational interventions. Preference surveys were administered, and audio recorded with 27 LTC residents, or family proxies, over the course of >70 sessions. Descriptive analysis of survey and audio data was conducted. Residents/families rated the majority (84%) of preferences on daily routines, social relationships, caregiving, and leisure as important; however, specific preferences endorsed were highly unique. Residents/families reported nearly half (42%) of preferences were rarely or never met and nearly all (94%) of their current preferences were not the same as before admission. Residents/families described negative impacts on mental well-being, quality of life, or physical health when preferences were not met and engaging in a process of adapting preferences to what was available at the facility. Findings highlight that both idiosyncratic and common preferences and care gaps exist across residents, and different interventions may be needed to address PCC at individual and organizational levels. Possible targets for operational interventions to address these different needs and improve PCC will be discussed.

